# Distinct properties of putative trophoblast stem cells established from somatic cell nuclear-transferred pig blastocysts

**DOI:** 10.1186/s40659-024-00516-y

**Published:** 2024-05-30

**Authors:** Eunhye Kim, Lian Cai, Hyerin Choi, Mirae Kim, Sang-Hwan Hyun

**Affiliations:** 1 Laboratory of Molecular Diagnostics and Cell Biology, College of Veterinary Medicine, Gyeongsang National University, Jinju, 52828 Republic of Korea; 2 Laboratory of Veterinary Embryology and Biotechnology, Veterinary Medical Center, College of Veterinary Medicine, Chungbuk National University, Cheongju, 28644 Republic of Korea; 3 Graduate School of Veterinary Biosecurity and Protection, Chungbuk National University, Cheongju, 28644 Republic of Korea; 4 Institute for Stem Cell & Regenerative Medicine (ISCRM), Lab. of Veterinary Embryology and Biotechnology (VETEMBIO), College of Veterinary Medicine, Chungbuk National University, 1 Chungdae-ro, Seowon-gu, Cheongju, 28644 Republic of Korea

**Keywords:** Trophoblast stem cells, Cloned pig, Somatic cell nuclear transfer, Placenta

## Abstract

**Background:**

Genetically modified pigs are considered ideal models for studying human diseases and potential sources for xenotransplantation research. However, the somatic cell nuclear transfer (SCNT) technique utilized to generate these cloned pig models has low efficiency, and fetal development is limited due to placental abnormalities.

**Results:**

In this study, we unprecedentedly established putative porcine trophoblast stem cells (TSCs) using SCNT and in vitro-fertilized (IVF) blastocysts through the activation of Wing-less/Integrated (Wnt) and epidermal growth factor (EGF) pathways, inhibition of transforming growth factor-β (TGFβ) and Rho-associated protein kinase (ROCK) pathways, and supplementation with ascorbic acid. We also compared the transcripts of putative TSCs originating from SCNT and IVF embryos and their differentiated lineages. A total of 19 porcine TSCs exhibiting typical characteristics were established from SCNT and IVF blastocysts (TSCs^NT^ and TSCs^IVF^). Compared with the TSCs^IVF^, TSCs^NT^ showed distinct expression patterns suggesting unique TSCs^NT^ characteristics, including decreased mRNA expression of genes related to apposition, steroid hormone biosynthesis, angiopoiesis, and RNA stability.

**Conclusion:**

This study provides valuable information and a powerful model for studying the abnormal development and dysfunction of trophoblasts and placentas in cloned pigs.

**Supplementary Information:**

The online version contains supplementary material available at 10.1186/s40659-024-00516-y.

## Background

The placenta is a unique transient organ that develops during pregnancy. It regulates maternal-fetal interactions, facilitates oxygen, nutrients, and waste exchange [1], provides immune protection against various infections, and supports fetal growth [2, 3]. However, placental abnormalities frequently occur in cloned fetuses produced by somatic cell nuclear transfer (SCNT), a technique successfully applied in agriculture, biomedicine, and translational research in regenerative medicine [4, 5]. Although pigs are considered ideal large animal models for human diseases [6–8], most implanted SCNT-derived porcine fetuses fail to develop to term [9, 10] owing to placental insufficiency [11, 12], stillbirth [13], and neonatal mortality [14]. However, the causes of placental abnormalities in cloned porcine fetuses remain unclear.

The development of early pre-implantation embryos differs among species [15]. In pigs, placental development begins at the morula stage, with the formation of the inner cell mass (ICM) and surrounding trophectoderm (TE) precursors [16, 17]. Porcine blastocysts expand into spherical, tubular, and filamentous structures before implantation. TE precursors differentiate into trophoblasts that initially support nutrient transfer and then form the diffuse epitheliochorial placenta [18, 19]. TE precursors in porcine blastocysts can proliferate without immortalization, and stem cells from TE precursors can be isolated and maintained in vitro as trophoblast stem cells (TSCs). Several putative porcine TSCs have been established from pre-implanted or implantation blastocysts [20–22]. Although in vitro-derived porcine trophoblast cells have been reported using in vitro fertilization (IVF) and parthenogenetic activation (PA) [23], porcine SCNT embryo-derived TSCs remain unreported.

Herein, we established and characterized putative TSCs from porcine blastocysts produced by IVF and SCNT. These TSCs retained their capacity to grow in culture without any immortalization procedure. We cultured them in a novel, optimized porcine-defined medium (pTS-medium), which supports the long-term self-renewal of independent TSCs derived from porcine blastocysts. Furthermore, we compared the characteristics of SCNT- and IVF-derived TSCs.

## Results

### Derivation of porcine TSCs from IVF and nuclear transfer (NT) blastocysts in a modified TS medium

**Fig. 1:**
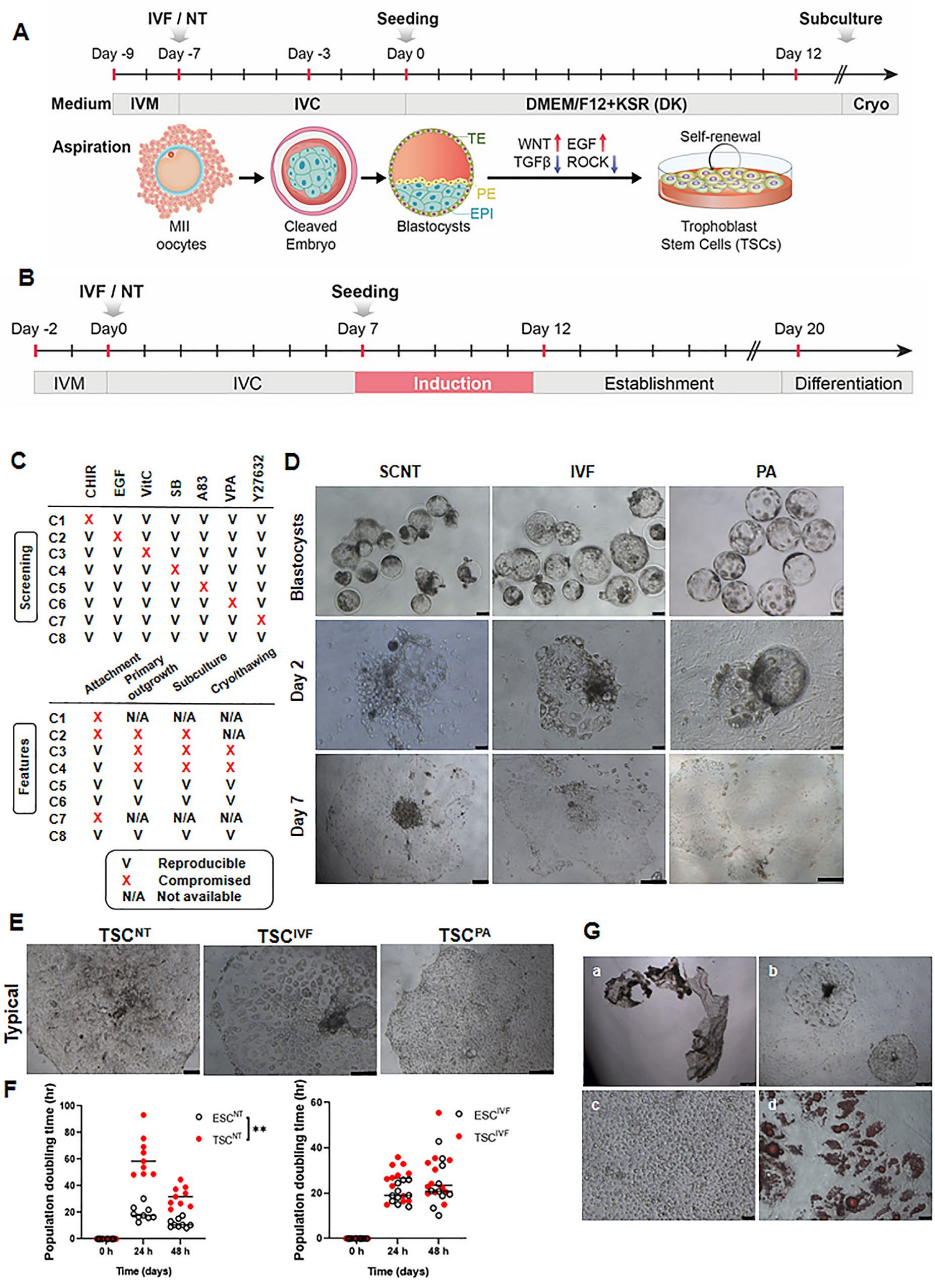
Derivation and maintenance of putative porcine trophoblast stem cells (TSCs) from various embryonic origins in pigs. (**A**) Schematic of porcine TSCs derivation from porcine embryos (**B**) Protocol from day 7 to day 12 for inducing porcine TSCs. (**C**) Screening of growth factors and inhibitors for deriving porcine TSCs. Table outlining tested media conditions with and without specific growth factors and inhibitors (top), and features observed from each condition (bottom). Ticks and crosses indicate included and excluded factors, respectively. NA, not applicable. CHIR, CHIR99021; SB, SB431542; A83, A83-01; VPA, valproic acid. (**D**) Morphological changes during TSC induction from porcine NT, IVF, and PA blastocysts (Scale bars = 100 μm). Representative morphologies of attached porcine NT/IVF/PA blastocysts 2 days after seeding (Scale bars = 50 μm) and expanded outgrowth 7 d after seeding (Scale bars = 200 μm) in novel pTS-medium. (**E**) Representative image of a typical colony of porcine TSCs derived from SCNT, IVF, and PA blastocysts. (Scale bars = 200 μm). (**F**) Population doubling (PD) time analysis of embryonic stem cell (ESC) and TSC colonies derived from porcine NT and IVF blastocysts. Data were analyzed by one-way ANOVA. **P < 0.01 (**G**) Maintenance of TSCs^PA^. Detached clumps derived from primary TSC colony derived from PA blastocysts (a) were attached 2 d after subculture (b). Accumulation of lipid droplet (c) in TSCs^PA^ was confirmed using oil red O staining (d). Scale bars = 200 μm

**Fig. 2:**
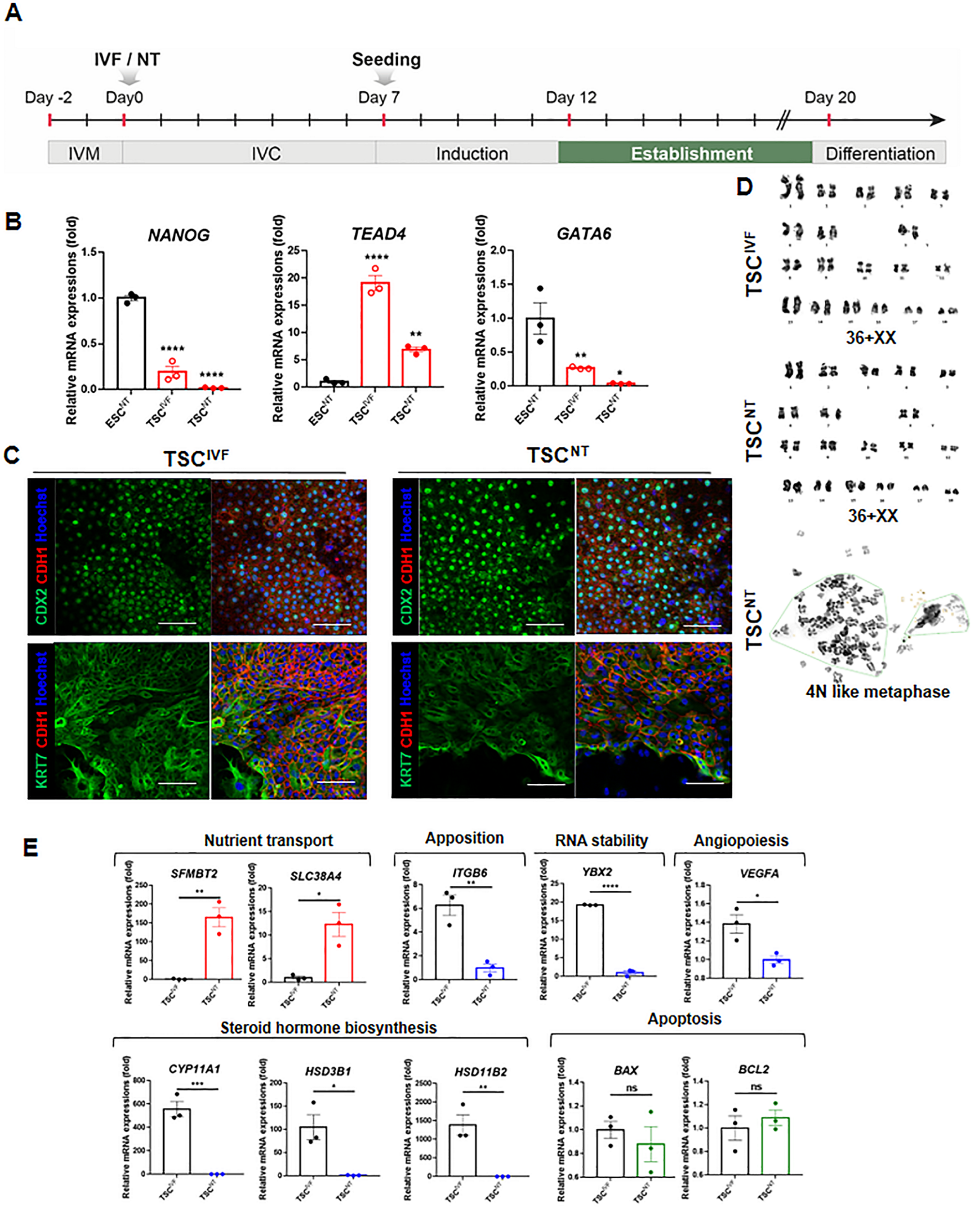
Characterization of putative porcine trophoblast stem cells (TSCs). (**A**) Protocol from day 12 to day 20 for establishing and characterizing porcine TSCs. (**B**) Relative mRNA expression levels of genes (*NANOG*, *GATA6*, and *TEAD4*) in TSC^IVF^ and TSC^NT^. Mean ± SEM; *n* = 3 for each group. Data were analyzed by one-way ANOVA. *P < 0.05, **P < 0.01, ***P < 0.001, and ****P < 0.0001. **(C**) The expression of trophoblast stem cell markers such as CDX2, KRT7, and E-cadherin (CDH1) in putative porcine TSCs was assessed by immunofluorescence. Scale bars = 100 μm. (**D**) Karyotyping of porcine TSCs. Normal chromosomes were observed in TSCs^IVF^ (36 XX) and TSCs^NT^ (36 XX). 4 N-like metaphase was also observed in TSCs^NT^. (**E**) Relative quantities of gene expression profiling data using real-time qPCR analysis of TSCs^NT^. Relative mRNA expression levels of genes (*ITGB6*, *YBX2*, *VEGFA*, *CYP11A1*, *CYP19A1*, *HSD3B1*, *HSD11B2, SFMBT2*, *SLC38A4*, *BAX*, *BCL2*,) were examined in TSC^IVF^ and TSC^NT^. Mean ± SEM; *n* = 3 for each group. Data were analyzed by t-tests. *P < 0.05, **P < 0.01, ***P < 0.001 and ****P < 0.0001

Porcine TSCs were derived from day 7 porcine blastocysts using a modified porcine TS medium (Fig. 1A). To induce TSCs (Fig. 1B), we screened several growth factors and inhibitors required for the proliferation and culture of various epithelial stem cells [24] and TSCs in other species (Fig. 1C, Additional file 1.) [25, 26]. We found that C5, C6, and C8 were optimal for the attachment, cell survival, subculture, and cryopreservation/thawing of our porcine TSCs. A total of 19 TSCs were established from porcine NT and IVF blastocysts (TSCs^NT^ and TSCs^IVF^; Table 1). Based on the order of the development, the 9 TSC primary colonies derived from NT blastocysts were named porcine TSC^NT^1-9, and the 10 TSC primary colonies grown from IVF blastocysts were named porcine TSC^IVF^1-10. During derivation, 2 days after seeding of porcine SCNT, IVF, and PA blastocysts, TS-like clones formed containing two cell morphologies: small round cells and giant cells originating from the trophectoderm (Fig. 1D). After 7 days of culture, primary cell colonies of TSCs^NT^, TSCs^IVF^, and TSCs^PA^ were formed. After 10–12 d of culture, the TS-like primary colonies were mechanically detached and dissociated. In porcine TS medium, subcultured colonies of the TSCs^NT^, TSCs^IVF^, and TSCs^PA^ showed the typical morphology of TSCs (Fig. 2A), including the formation of a tight epithelial cell-like morphology with bright boundaries. During TSC derivation, the attachment and outgrowth rates of porcine TSCs were significantly higher in the treatment group (porcine novel TS medium) than in the control group (conventional porcine TS medium reported by Hou et al. [23]) in both TSCs^NT^ and TSCs^IVF^ (Table 2). The efficiency of establishing porcine TSCs in the novel porcine TS medium was also higher than that of the control in both NT and IVF embryos (Table 2). These results suggest that putative porcine TSCs can be efficiently derived from porcine NT and IVF blastocysts using the porcine TS medium. In general, the calculated population doubling time (PD time) of porcine TSCs was longer than that of porcine embryonic stem cells (ESCs) reported previously [27], regardless of their origin (NT and IVF blastocysts) (Fig. 2B). The ESC^NT^ and ESC^IVF^ lines grew with a PD time of approximately 15.4 h and 21.9 h, respectively. In contrast, TSCs^NT^ and TSCs^IVF^ duplicated their population approximately once in 47.1 h and 26.5 h, respectively. All TSCs^NT^ and TSCs^IVF^ derived from porcine blastocysts exhibited similar morphology during maintenance. However, interestingly, the TSCs^PA^ showed different morphologies during maintenance, retaining a higher accumulation of lipid droplets (observed using Oil Red O staining) making it difficult to culture on a long-term, distinct from the TSCs^NT^ and TSCs^IVF^ (Fig. 2C). In this study, we focused on features of abnormal development and dysfunction of trophoblasts and placentas in cloned pigs produced using the SCNT technique, and thus comparatively analyzed SCNT-derived TSCs (TSCs^NT^) with IVF-derived TSCs (TSCs^IVF^) as a control. Therefore, subsequent experiments focused on the TSCs^NT^ and TSCs^IVF^, excluding the TSCs^PA^.

**Table 1:** Information of established putative porcine trophoblast stem cells

Cells	Origins (porcine)	Initial culture methods	Medium condition	Day of seeding	Passaging
**TSC** ^**NT**^ **_1**	NT blastocysts	Whole explant	Novel	Day 7	More than 30
**TSC** ^**NT**^ **_2**	NT blastocysts	Whole explant	Novel	Day 7	More than 30
**TSC** ^**NT**^ **_3**	NT blastocysts	Whole explant	Novel	Day 7	More than 30
**TSC** ^**NT**^ **_4**	NT blastocysts	Whole explant	Novel	Day 7	More than 25
**TSC** ^**NT**^ **_5**	NT blastocysts	Whole explant	Novel	Day 7	More than 25
**TSC** ^**NT**^ **_6**	NT blastocysts	Whole explant	Conventional	Day 7	More than 18
**TSC** ^**NT**^ **_7**	NT blastocysts	Whole explant	Conventional	Day 7	More than 18
**TSC** ^**NT**^ **_8**	NT blastocysts	Whole explant	Novel	Day 7	More than 17
**TSC** ^**NT**^ **_9**	NT blastocysts	Whole explant	Novel	Day 7	More than 17
**TSC** ^**IVF**^ **_1**	IVF blastocysts	Whole explant	Novel	Day 7	More than 21
**TSC** ^**IVF**^ **_2**	IVF blastocysts	Whole explant	Conventional	Day 7	More than 21
**TSC** ^**IVF**^ **_3**	IVF blastocysts	Whole explant	Novel	Day 7	More than 21
**TSC** ^**IVF**^ **_4**	IVF blastocysts	Whole explant	Novel	Day 7	More than 21
**TSC** ^**IVF**^ **_5**	IVF blastocysts	Whole explant	Novel	Day 7	More than 20
**TSC** ^**IVF**^ **_6**	IVF blastocysts	Whole explant	Conventional	Day 7	More than 17
**TSC** ^**IVF**^ **_7**	IVF blastocysts	Whole explant	Novel	Day 7	More than 17
**TSC** ^**IVF**^ **_8**	IVF blastocysts	Whole explant	Novel	Day 7	More than 15
**TSC** ^**IVF**^ **_9**	IVF blastocysts	Whole explant	Novel	Day 7	More than 15
**TSC** ^**IVF**^ **_10**	IVF blastocysts	Whole explant	Novel	Day 7	More than 15

*Abbreviations* TSC, trophoblast stem cell; NT, nuclear transfer; IVF, in vitro fertilization

**Table 2:** Efficiency of putative porcine trophoblast stem cells derived from blastocysts

Embryo origins	Group	No. of blastocysts		No. of primary outgrowths, n (%)	No. of cell lines, n (%)
		Plated	Attached, *n* (%)		
NT	Conventional	21	8 (37.1 ± 5.3)	4 (17.5 ± 6.9)	2 (8.3 ± 4.8)
	Novel	21	17 (80.4 ± 2.0)*	11 (52.5 ± 2.5)*	7 (32.1 ± 6.6)*
	Total	42	25	15	9
IVF	Conventional	27	11 (40.7 ± 2.8)	6 (23.4 ± 6.1)	2 (7.5 ± 7.2)
	Novel	27	21 (81.5 ± 6.1) *	15 (54.6 ± 10.3) *	8 (29.0 ± 4.2) *
	Total	54	32	21	10

*Abbreviations* NT, nuclear transfer; IVF, in vitro fertilization. Data were analyzed by t-tests. *P < 0.05

### Characterization of the established porcine TSCs^NT^ and TSCs^IVF^ derived from blastocysts

**Fig. 3:**
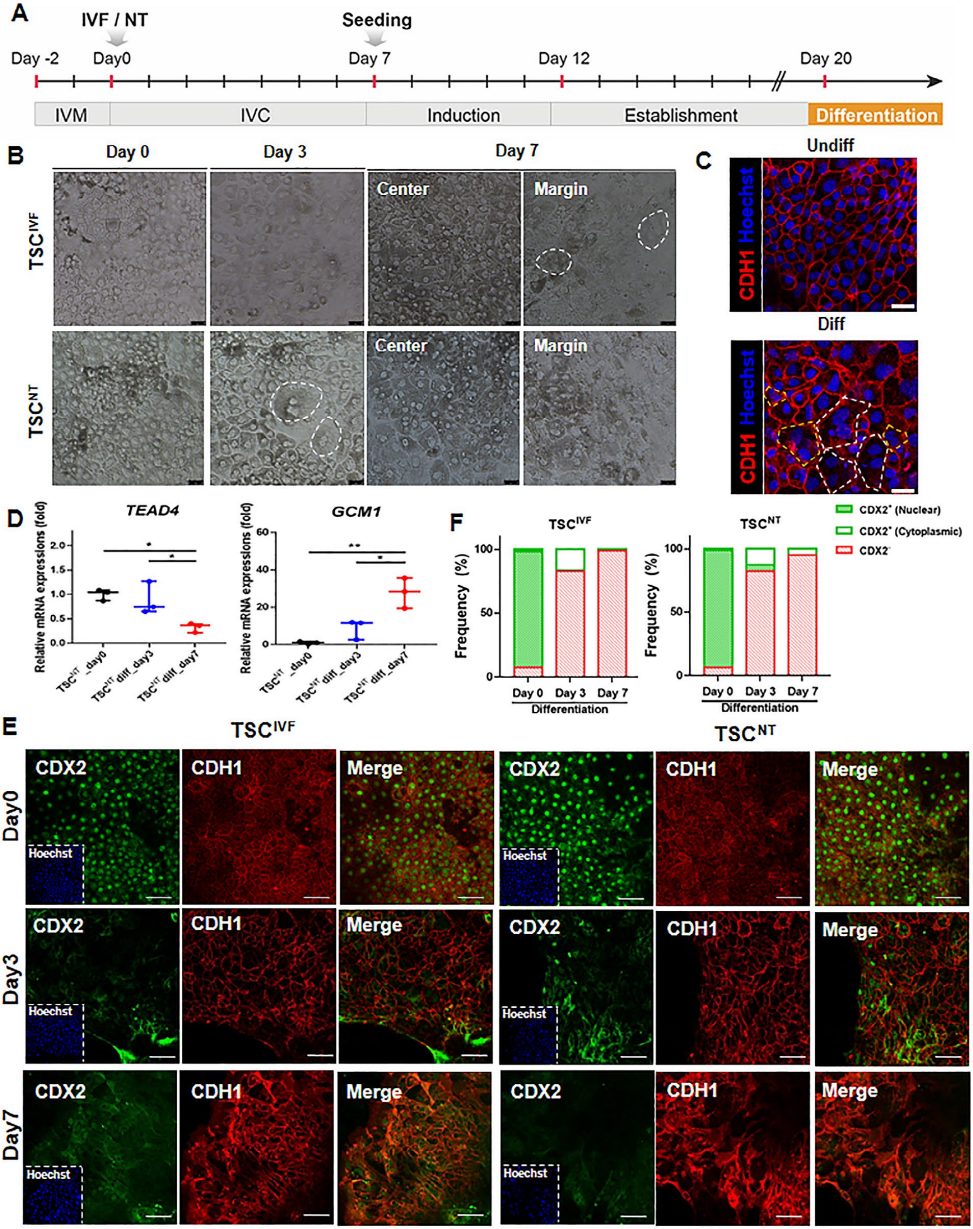
Differentiation capacity of putative porcine TSCs from IVF and NT blastocysts. (**A**) Differentiation step of porcine TSCs. (**B**) Morphological changes of TSCs during differentiation (Day 0, Day 3, and Day 7). Scale bars = 100 μm. (**C**) E-cadherin (CDH1) expression in differentiated putative porcine TSCs^NT^ assessed by immunofluorescence showing limited multinucleated trophoblasts containing three (white dotted line) or two (yellow dotted line) nuclei. Scale bars = 100 μm. (**D**) Relative mRNA expression levels of genes (*TEAD4* and *GCM1*) in differentiated TSC^NT^. Mean ± SEM; *n* = 3 for each group. Data were analyzed by one-way ANOVA. *P < 0.05 and **P < 0.01. (**E**) CDX2 and E-cadherin (CDH1) expression in TSCs^IVF^ and TSCs^NT^ during differentiation (Day 0, Day 3, and Day 7). Scale bars = 200 μm. (**F**) Quantified frequency of CDX2^+^ cells in TSCs^IVF^ and TSCs^NT^ during differentiation

Next, we investigated and identified the characteristics of the porcine TSCs^NT^ and TSCs^IVF^ (Fig. 3A) during passages 15–17. We used qRT-PCR to examine the mRNA expression levels of important markers involved in early lineage specification during porcine embryogenesis (Fig. 3B) in porcine putative TSCs compared to those of porcine ESCs previously established by our group [27]. The expression of a core transcription factor of porcine pluripotent epiblast, *Nanog* [17, 29], decreased in both TSCs and increased in the ESC lines. Both TSCs displayed significantly increased mRNA expression of the *TEAD4* gene, which is a TSC marker [28] and involved in TE specification [17, 29], compared to the ESC lines. Additionally, the expression level of the earliest marker of the primitive endoderm (PrE), *GATA6* [30], was rarely detected in either TSCs compared to ESC lines. These results indicate that the TSCs do not contain porcine ICM or PrE lineage cells.

To validate these results and identify TSC characteristics at the protein level, immunofluorescence analysis was performed. We observed significant expression of intracellular CDX2, a TSC-specific marker [31], in both the TSCs^NT^ and TSCs^IVF^ (Fig. 3C). Additionally, cytokeratin 7 (KRT7), a representative pan-trophoblast marker [32], was distinctly expressed in the cytoplasm of both porcine TSCs^NT^ and TSCs^IVF^. The intercellular junctions were characterized by the expression of the E-cadherin CDH1, a transcription factor in epithelial cells [33]. Karyotyping of the TSCs^NT^ revealed that the representative cell line contained the normal number of 38 chromosomes after 30 passages (Fig. 3D). One of the three TSCs^NT^ had a 4 N-like metaphase with 76 chromosomes. Together, these experiments demonstrate that porcine TSCs from NT and IVF embryos in porcine TS medium have typical TSC characteristics similar to those of other species.

### Comparative analysis of relative mRNA expression in the established porcine TSCs^NT^ and TSCs^IVF^

The distinct properties of cloned piglet placentas could be explained by comparing the characteristics of porcine TSCs^NT^ against those of TSCs^IVF^. The placentas of cloned piglets showed severe trophoblast hypoplasia [34–36], which is related to nutrient transport, blastocyst apposition, RNA stability, angiopoiesis, steroid hormone biosynthesis, and apoptosis during implantation stages. Thus, we examined the related gene expressions between the TSC^NT^ and TSC^IVF^ groups. The mRNA expression of the nutrient transport-related genes *SFMBT2* and *SLC38A4* [37] was significantly higher in the porcine TSCs^NT^ group than in the TSCs^IVF^ group (Fig. 3E). Critical genes involved in opposing porcine blastocysts during implantation, such as *ITGB6* [38], were significantly downregulated only in the TSCs^NT^ group. Additionally, the expression of *YBX2*, a transcriptional regulator of RNA stability [39], was significantly lower in the porcine TSCs^NT^ group than in the TSCs^IVF^ group. Placental abnormalities in cloned animals include reduced vascularization [40, 41]. The key transcription factor for vasculogenesis and angiopoiesis during placental development, *VEGFA* [42], was downregulated in TSCs^NT^ compared to TSCs^IVF^. The *CYP11A1*, *HSD3B1*, and *HSD11B2* mRNA expression levels were significantly lower in the TSCs^NT^ than in the TSCs^IVF^ (Fig. 3E), suggesting that the TSCs^NT^ group had a diminished steroid hormone biosynthesis capacity than the TSC^IVF^ group. No significant differences existed in the expression of the apoptosis-related genes *BAX* and *BCL2* between the TSC^NT^ and TSC^IVF^ groups.

### Differentiation of the established porcine TSCs^NT^ and TSCs^IVF^

**Fig. 4:**
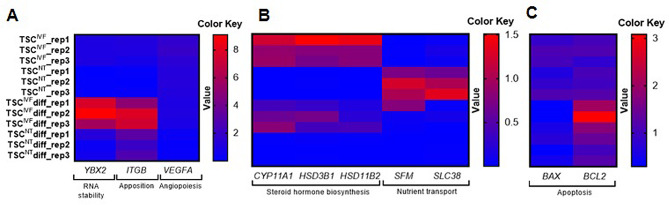
Relative quantities of mRNA expression profiling data using real-time qPCR analysis of differentiated TSC^IVF^ versus TSC^NT^. Heat map comparing the expression of transcription factors of RNA stability, apposition, angiopoiesis, steroid hormone biosynthesis, nutrient transport, and apoptosis in TSCs. Red and blue represent genes with high and low expression levels, respectively. TSC **r**ep1, rep2, and rep3 are three biological replicates of TSC RNA samples

Next, we investigated the differentiation capacity of the established porcine TSCs (Fig. 4A). When the undifferentiated TSCs grown in feeder cells were removed from the coculture conditions and grown without porcine novel TS medium, they spontaneously differentiated. Under these conditions, both the TSC^NT^ and TSCs^IVF^ showed dramatic morphological changes (Fig. 4B). By the third day of differentiation, the porcine TSCs^NT^ displayed a fused trophoblast giant cell (TGC)-like morphology (Fig. 4B, white dotted line). On day 7, TGC-like morphology was observed in the marginal area of the TSC^IVF^ group. These fused forms of the TGC-like morphology in the differentiated group were also observed using CDH1 immunostaining and showed limited multinucleated trophoblasts containing up to three nuclei (Fig. 4C, Additional file 2). We found that the mRNA expression of *TEAD4*, critical for trophoblast progenitor self-renewal [43], was significantly lower on day 7 after differentiation induction than that in the undifferentiated TSC^NT^ and day 3-differentiated groups (Fig. 4D). More importantly, the mRNA expression of *GCM1*, a TSC differentiation-associated transcript [44], was greater on day 7 after differentiation induction than those in undifferentiated TSC^NT^ and day 3-differentiated groups. In addition, we observed a localization change in CDX2 expression after differentiation using immunofluorescence (Fig. 4E). Although some cells maintained CDX2 expression in the nucleus on day 3, almost all of this enrichment was evenly distributed within the cytoplasm and was absent in the nucleus on day 7 (Fig. 4F). This result likely indicates compromised expression of representative TE-specific markers for porcine TSC differentiation.

**Fig. 5:**
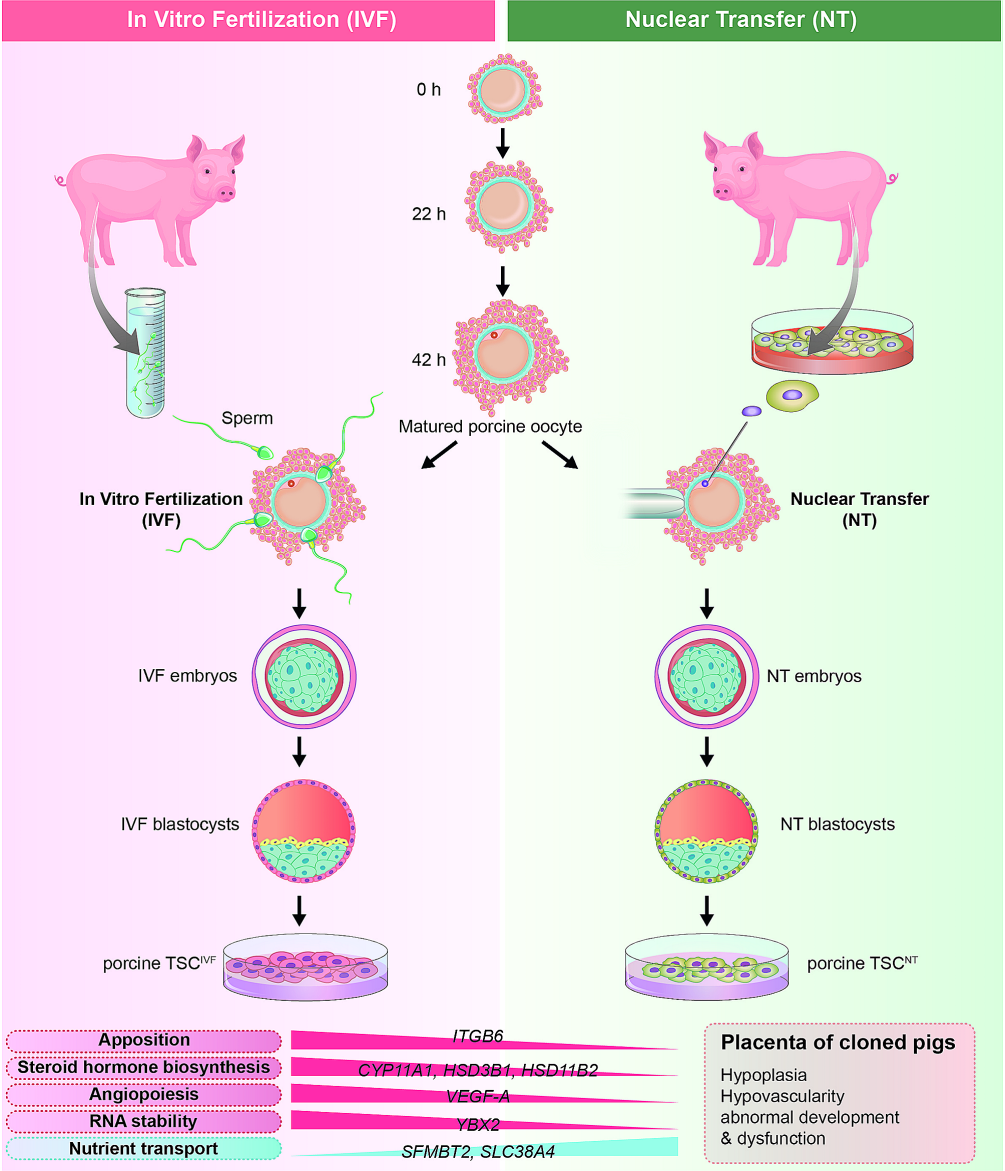
Schematic view of porcine TSC^IVF^ and TSC^NT^. Porcine TSC^NT^ retain the distinct properties related to apposition, steroid hormone biosynthesis, angiopoiesis, and RNA stability compared to porcine TSC^IVF^, potentially leading to placental hypoplasia and hypovascularity and further abnormal development and dysfunction in cloned piglets

Finally, we sought to characterize the changes in the relative mRNA expression level after the porcine TSCs^NT^ and TSCs^IVF^ differentiated using real-time qPCR analysis. Compared to the undifferentiated group, *YBX2* and *ITGB* were upregulated in the differentiated TSC^IVF^ group, as illustrated in the heat map (Fig. 5). Interestingly, steroid hormone biosynthesis-related mRNAs (*CYP11A1*, *HSD3B1*, and *HSD11B2*) were upregulated in the TSC^IVF^ group compared to the TSC^NT^ group under both undifferentiated and differentiated conditions. The upregulation of nutrient transport-related genes (*SFM* and *SLC38*) in undifferentiated TSC^NT^ was abrogated in differentiated TSC^NT^. The differentiated TSC^IVF^ group showed upregulated BCL2 expression compared to the other groups. Overall, compared with the TSC^IVF^ groups, the distinct expression patterns of the undifferentiated and differentiated TSC^NT^ groups suggested unique characteristics of the TSCs derived from porcine SCNT embryos.

## Discussion

Herein, we developed putative porcine TSCs from porcine SCNT blastocysts cultured in our novel medium, creating an in vitro model for trophoblast differentiation. We further revealed that porcine TSCs^NT^ and TSC^IVF^ share similar features, including morphological characteristics and capacity for differentiation, but showed distinct gene expression profiles possibly associated with placental abnormalities in cloned piglets (Fig. 5).

Placental hyperplasia, known as placentomegaly, is the most prominent characteristic of cloned mice [45, 46]. The placentas of cloned mouse conceptuses are two- to five-fold larger than control conceptuses. These abnormalities are thought to be associated with the upregulation of nutrient transport-related genes such as *SFMBT2* and *SLC38A4* in SCNT mice [47]. In contrast, the placentas of cloned piglets possessed low villus density and exhibited severe trophoblast hypoplasia [34–36]. Therefore, we expected these genes to be downregulated in the TSCs^NT^ of the cloned pigs with placental hypoplasia. Unexpectedly, the mRNA expression of *SFMBT2* and *SLC38A4* was significantly higher in the TSCs^NT^ group than in the TSCs^IVF^ group, similar to other mice TSCs with placentomegaly [37]. These results suggest that other factors may be more critical than nutrient transport, causing abnormalities in the implantation and development of the cloned porcine placenta.

The initial stage of placentation during the adhesion cascade for implantation differs across species [48]. The key events during porcine conceptus implantation include (1) hatching from the zona pellucida and rapid elongation, (2) pre-contact with the uterine luminal epithelium (LE) and orientation of the blastocyst, (3) apposition of the trophectoderm to the endometrial LE followed by unstable adhesion, and (4) development of interdigitating microvilli between the trophectoderm and uterine LE [49]. After the rapid elongation step, porcine conceptuses secrete estrogen, a steroid hormone primarily incorporated into the mammalian conceptus implantation [50]. In the placental transcriptome data of Ao et al. [51], steroid hormone biosynthesis enzymes were downregulated in porcine SCNT placentas compared to their artificially inseminated counterparts. We found the mRNA expression of steroid hormone biosynthesis enzymes, including *CYP11A1*, *HSD3B1*, and *HSD11B2* to be downregulated in the TSCs^NT^ compared to that in the TSCs^IVF^. Therefore, downregulated steroid hormone-related levels in SCNT TSCs may cause low viability of SCNT pig fetuses. Furthermore, the secreted estrogen induces the synthesis and secretion of osteopontin (OPN) in the uterine LE of pigs [38]. OPN then binds integrin heterodimers αv (ITGAV) and β6 (ITGB6) on the trophectoderm and αv and β3 (IGTB3) on the uterine LE [52] to bridge conceptus apposition to the uterine LE for implantation. In the present study, the expression of *ITGB6*, an integrin subunit in the trophectoderm, was downregulated in porcine TSCs^NT^ relative to that in TSCs^IVF^, and this pattern was retained even in differentiated TSCs^NT^. Therefore, decreased *ITGB6* expression may impair the development of cloned porcine conceptuses during the peri-implantation period.

In mammals, the placenta is a highly vascularized transient organ with diverse placentation types [53]. Pigs have a diffuse epitheliochorial placenta, which is less invasive; therefore, they have layers of maternal tissue separating the fetus from the maternal blood [54, 55]. Although placental vascularization is critical for nutrient transport from the mother to the fetus [56], cloned porcine fetuses display villous hypovascularity [36]. We found that the mRNA of the vascular endothelial growth factor *VEGF-A*, the most critical factor for placental vascularization [42], was poorly expressed in porcine TSCs^NT^ compared to that in TSCs^IVF^. This suggests that reduced placental vascularization and abnormalities in cloned pigs might be due to a lack of *VEGF-A* expression.

Standardized media are crucial in stem cell research [57, 58]. Herein, we determined the minimum requirements for porcine TSCs. Thus, porcine TSCs derived from SCNT and IVF embryos can be efficiently maintained without FBS in media supplemented with GSK3, TGFβ, ROCK inhibitors, EGF, and L-ascorbic acid without losing their stemness, self-renewal, and differentiation capabilities. The novel culture conditions for porcine TSCs in this study are substantially different from those reported by Hou et al. [23], the only common ingredient being the ROCK inhibitor, Y27632. This novel media significantly increased attachment, primary outgrowth rate, and TS cell line establishment compared to conventional media [23]. A specific culturing system, containing Epidermal Growth Factor (EGF), reinforcing the WNT pathway, and inhibiting the TGFβ signaling, was successfully used to derive human TSCs from embryos [59]; however, its effects on in vitro cultured porcine trophoblast stem cells have not been investigated so far. Our study demonstrated that the porcine TSC lines could be obtained from SCNT embryos by controlling WNT/TGFb signaling. However, A83-01, another TGFβ receptor inhibitor [60] like SB431542, and valproic acid (VPA), a histone deacetylase inhibitor, which is known to enhance the proliferation of human villous cytotrophoblast cells [25], were not essential for culture and maintenance of porcine TSCs.

The dynamic transition of epithelial cells to motile mesenchymal cells, known as the epithelial-mesenchymal transition (EMT), occurs in diverse developmental processes [61, 62]. Trophectoderm differentiation for initiating placental formation is the first developmental EMT [63]. One of the molecular events involved in the initiation and completion of EMT is the loss of the adhesive cell-surface protein E-cadherin (CDH1), required to form intercellular junctions in polarized cells [64]. Despite prolonged culture, our porcine TSC lines maintained apical-basal polarity with E-cadherin expression, a hallmark of epithelial cells [64]. After 7 days of differentiation, CDH1 expression receded from the periphery of TSC lines, transitioning epithelial cells into CDH1-lacking mesenchymal cells. This indicates the initial EMT event for placental development, consistent with other reports on human TSCs [65]. However, unlike human TSCs, porcine-differentiated TSCs in our study showed limited multinucleated trophoblasts containing up to three nuclei. This aligns with the fact that animals with epitheliochorial placentation, such as pigs and horses, do not form a syncytiotrophoblast layer; however, binucleate or trinucleate cells are present in the placenta [66]. The expression of *GCM1*, a key transcription factor classically associated with syncytiotrophoblast formation [44], was increased on day 7. Furthermore, in our study, we found that nuclear CDX2 expression in porcine TSCs disappeared during differentiation. This is most likely due to the result of the loss of CDX2 expression during the differentiation of TSCs into more committed trophoblast lineages in extraembryonic tissues [67]. However, a limitation of this study is that analysis of the in vivo differentiation capacity of porcine TSCs remains insufficient. Although conducting a further study like blastocyst injection-mediated placental chimerism or comparative RNA sequencing might provide stronger data to demonstrate its applicability as an in vitro model of TSCs, the current study provides preliminary insights into the fundamental studies of comparative cellular physiology of porcine TSCs derived from NT blastocyst compared to IVF blastocysts.

## Conclusion

Overall, compared with the TSC^IVF^ groups, the distinct expression patterns of the undifferentiated and differentiated TSC^NT^ groups suggested unique characteristics of TSC^NT^, which might provide insights into the specific molecular and cellular features of abnormal development and dysfunction of trophoblasts and placentas in cloned pigs. In addition, we believe that the putative porcine TSCs established in this study might provide cell sources for the molecular and functional analyses of porcine trophoblast lineages and the generation of porcine blastoids.

## Materials and methods

### Ethics statement

This study followed the Guide for the Care and Use of Laboratory Animals of the National Veterinary and Quarantine Services. The study protocol was approved by the Committee on the Ethics of Animal Experiments of Chungbuk National University (Permit Number: CBNUA-1460-20-02).

### Chemicals

Unless otherwise indicated, all chemicals and reagents used in this study were purchased from Sigma-Aldrich Corporation (St. Louis, MO, USA).

### Oocyte collection and in vitro maturation (IVM)

Ovaries of prepubertal gilts were collected in 0.9% (w/v) saline solution supplemented with 100 IU/L penicillin G and 100 mg/mL streptomycin sulfate, maintained at a temperature range from 32 to 35 ˚C at a local abattoir, and transported to the laboratory within 2 h after collection. Cumulus oocyte complexes (COCs) were aspirated from superficial follicles with an 18-gauge needle, allowed to sediment in 15 mL conical tubes at 37 ˚C for 5 min, and resuspended and washed several times with HEPES-buffered Tyrode’s medium (TLH) containing 0.05% (w/v) polyvinyl alcohol (TLH-PVA). Using a stereomicroscope, we selected only COCs having ≥ 3 uniform layers of compact cumulus cells and a homogenous cytoplasm. In each well of a four-well plate (Nunc Thermo Scientific, Leicestershire, UK), we cultured approximately 60 COCs in 500 µL of IVM medium (TCM199; Invitrogen Corporation, Carlsbad, CA, USA) supplemented with 0.6 mM cysteine, 0.91 mM sodium pyruvate, 10 ng/mL epidermal growth factor, 75 µg/mL kanamycin, 1 µg/mL insulin, 10% (vol/vol) porcine follicular fluid, 10 IU/mL equine chronic gonadotropin, and 10 IU/mL human chorionic gonadotropin (Intervet, Boxmeer, Netherland). Subsequently, the selected COCs were incubated at 39 ˚C, 5% CO_2_, and 95% humidity. After 21–22 h of hormone-induced maturation, the COCs were washed twice and cultured in fresh hormone-free IVM medium for an additional 21–22 h.

### Nuclear transfer (NT), fusion, and activation

After IVM, the COCs were denuded by gentle pipetting with 0.1% hyaluronidase, washed three times with TLH-PVA, incubated for 5 min in manipulation medium (calcium-free HEPES-buffered Tyrode’s medium containing 0.4% (w/v) bovine serum albumin) with 5 mg/mL Hoechst 33,342, rinsed twice with fresh manipulation medium, transferred into a drop of manipulation medium containing 5 mg/mL cytochalasin B, and enucleated by aspirating the polar body and MII chromosomes using a 16-mm glass pipette (Humagen, Charlottesville, VA, USA). After enucleation, trypsinized donor cells with smooth cell surfaces were transferred into the perivitelline space of the enucleated oocytes using a fine injection pipette. The couplets were placed in a fusion/activation medium (280 mM mannitol, 1.0 mM CaCl_2,_ and 0.05 mM MgCl_2_, and pH 7.0–7.4), and electrical fusion/activation was performed using a cell fusion generator and two 60 µs electrical pulses of 160 V/mm (LF101; NepaGene, Chiba, Japan). After 30 min, the fusion rate was evaluated, and oocytes were treated for 4 h with 2 mM 6-dimethylamino purine and 0.4 mg/mL demecolcine in the IVC medium at 39 ˚C in a humidified atmosphere containing 5% O_2_, 5% CO_2_, and 90% N_2_.

### In vitro fertilization (IVF) and parthenogenetic activation (PA) of oocytes

For IVF, 15 denuded oocytes at the MII stage were transferred in 40 µL modified Tris-buffered medium (mTBM) [68] in a 35 × 10 mm Petri dish (Falcon; Becton Dickinson Labware, Franklin Lakes, NJ, USA) covered with pre-warmed mineral oil. Fresh liquid porcine semen supplied weekly from the Veterinary Service Laboratory (Department of Livestock Research, Yong-in City, Gyeonggi-do, Republic of Korea) was kept at 17 ˚C for 3 d before use. Semen was added to 10 mL warmed Dulbecco’s phosphate-buffered saline (PBS) supplemented with 0.1% BSA, and the mixture was centrifuged at 2000 × g for 2 min. After washing twice, the sperm pellet was suspended in mTBM pre-equilibrated for 18 h at 39 ˚C in a 5% CO_2_ atmosphere. After an appropriate dilution, 5 µL of the sperm suspension was added to the 40-µL oocyte drops at a final concentration of 1 × 10^6^ sperm/mL, and incubated for 20 min at 39˚C in a humidified atmosphere containing 5% CO_2_ and 95% air. After removing the loosely attached sperm from the zona pellucida by gentle pipetting, the oocytes were washed and incubated in mTBM without sperm for 5 h at 39 ˚C in a humidified atmosphere containing 5% CO_2_ and 95% air. For PA, experiments were performed as previously described [69]. In brief, denuded oocytes at the MII stage were rinsed twice with the activation medium and placed between electrodes covered with the activation medium in a chamber connected to an electrical pulsing machine (LF101; NepaGene, Chiba, Japan). The oocytes were then activated with two direct-current pulses of 120 V/mm for 60 µs and immediately placed in porcine zygote medium 3 (PZM3) supplemented with 5 µg/mL cytochalasin B for 6 h. The embryos were cultured in 25 µL micro-drops (10 gametes/drop) of PZM3 [70] at 39 ˚C for 168 h under a humidified atmosphere containing 5% O_2_, 5% CO_2_, and 90% N_2_. In all experiments, the embryo culture media were replaced at 48 h (Day 2) and 96 h (Day 4).

### Feeder cell preparation

Mouse embryonic fibroblasts (MEF) were used as feeders. To prepare feeder cells, fetal heads, internal organs, and legs were removed from embryonic day 13.5 ICR mouse fetuses. The remaining tissues were minced in PBS and centrifuged at 2000 rpm for 3 min at least twice. The resulting MEFs were cultured in Dulbecco’s Modified Eagle Medium (DMEM; Gibco, Carlsbad, CA, USA) with 10% FBS (Gibco, Carlsbad, CA, USA), 1% non-essential amino acids (Gibco, Carlsbad, CA, USA), 1% glutamine (Gibco, Carlsbad, CA, USA), 0.1 mM ß-mercaptoethanol (Gibco, Carlsbad, CA, USA), and 1% antibiotic-antimycotic (Gibco, Carlsbad, CA, USA) (growth medium) at 37 ˚C in a 5% CO_2_ atmosphere. MEFs at passages 2–3 were inactivated with 10 µg/mL mitomycin C (Roche, Basel, Switzerland) for 2–2.5 h, plated at a density of 5 × 10^5^ cells/mL in a 4-well dish coated with 0.5% gelatin in growth medium, and used to seed porcine TSCs.

### Derivation, culture, and differentiation of putative porcine trophoblast stem cells (TSCs)

To remove the zona pellucida, porcine NT, IVF, and PA blastocysts were incubated with 0.5% protease for 1 min. For plating, intact blastocysts on day 7 were washed, plated directly onto mitomycin C-inactivated MEF feeder layers under a microscope, and cultured in a novel pTS-medium. This medium consisted of DMEM/F12 (Gibco, Carlsbad, CA, USA) containing 1% non-essential amino acids, 0.1 mM ß-mercaptoethanol, 1% antibiotic-antimycotic and 20% KOSR (Gibco) supplemented with 2 µM CHIR99021 (GSK3i; CT99021, Selleck Chemicals, Houston TX), 2 µM transforming growth factor-β (TGFβ) receptor inhibitor SB431542 (Sigma, S4317), 10 ng/mL epidermal growth factor (EGF), 10 µM Y27632, and 250 µM L-ascorbic acid. After 48 h, the attachment efficiency of primary cultures was determined by scoring the number of attached colonies. Following 5–7 d of culture, TS-like primary colonies were derived and designated as passage zero (P0). Primary TS-like cell colonies were mechanically dissociated into several clumps using pulled glass pipettes under a stereomicroscope. The clumps were re-seeded onto fresh inactivated MEFs. Subsequent TSCs were passaged mechanically every 6–8 d, and the medium was changed daily. All TSCs were cultured at 37˚C in an atmosphere with 5% CO_2_ and controlled humidity. To investigate the differentiation capacity of the putative TSCs, we induced spontaneous differentiation by removing the feeder cells and porcine novel TSC medium condition. All differentiated TSCs were cultured at 37 ˚C in an atmosphere with 5% CO_2_ and controlled humidity.

### Gene expression analysis by real-time PCR

For gene expression analysis, all samples were washed in DPBS and stored at -80 ˚C. Total RNA from porcine embryonic stem cells (ESCs), TSCs, and MEFs was extracted using the TRIzol reagent (TaKaRa Bio Inc., Otsu, Shiga, Japan) following the manufacturer’s protocol. RNA was quantified and treated with a gDNA remover. Complementary DNA (cDNA) was prepared from the extracted mRNA as previously described [71]. The synthesized cDNA was mixed with 2× SYBR Premix Ex Taq (Takara Bio Inc.) for qRT-PCR (Mx3000P qRT-PCR; Agilent Technologies, Santa Clara, CA, USA) using 10 pmol of each primer. The primer sequences used for qRT-PCR are listed in Additional file 3. PCR reactions were performed for 40 cycles under the following conditions: denaturation at 95 ˚C for 15 s, annealing at 57 ˚C for 15 s, and extension at 72 ˚C for 15 s. Gene expression fold change was determined using the following equation: *R* = 2^−[ΔCt sample – ΔCt control]^. The quantitative levels of all genes were normalized to endogenous *GAPDH* values. All experiments were performed at least three times.

### Immunofluorescence (IF) analysis

IF was performed as follows: cells were fixed with 4% paraformaldehyde (PFA) for 10 min, incubated with blocking buffer (5% goat serum, 0.5% BSA, 0.25% Triton-X 100 in PBS) for 1 h, and labeled with primary antibodies (shown in Additional file 4.) overnight at 4 ˚C. The following day, cells were washed thrice with washing buffer (0.2% Tween-20 and PBS) and incubated with the appropriate secondary antibodies for 1 h at room temperature. For cytokeratin 7 (KRT7) imaging, after a 1-h incubation with biotinylated goat-anti-mouse F(ab’)2 IgG fragments (2.5 µg/mL), a TSA Green kit (Tyramide Signal Amplification; Perkin Elmer, www.perkinelmer.com, Waltham, MA) was used to enhance the immunostaining signal. Nuclei were stained with Hoechst 33,342 and mounted on Vectashield (Vector Labs, www.vectorlabs.com, Burlingame, CA). Stained cells were examined using a confocal microscope and the ZEN 2009 Light Edition software (Carl Zeiss, Oberkochen, Germany).

### Alkaline phosphatase (AP) staining

To detect AP activity, porcine TSCs were fixed with 4% paraformaldehyde for 5 min at room temperature and stained with a solution containing nitro blue tetrazolium chloride (NBT) and 5-bromo-4-chloro-3-indolyl phosphate toluidine salt (BCIP) chromagen solution (Roche, Basel, Switzerland). The stained cells were washed and analyzed under a microscope.

### Oil red O staining

The accumulation of lipid droplets was evaluated by Oil Red O staining. Briefly, the TSCs were washed with PBS and fixed with 4% (w/v) PFA for 5 min at room temperature. The cells were then stained with 5 µg/mL Oil Red O (Sigma) for 10 min and examined by light microscopy.

### Cell population doubling (PD) time

The population doubling (PD) time of the putative porcine TSCs was measured as previously described [27] and compared with the PD time of the porcine ESCs reported by our group in a study published in 2016 [27]. Briefly, colonies were evaluated 2 d after plating. Longitudinal and horizontal diameters were measured for each colony using ImageJ (NIH, Bethesda, MD, USA). The approximate colony area was calculated using the surface area equation of an ellipse (π × a×b/4, where a and b are the horizontal and longitudinal diameters, respectively).

### Karyotyping

To induce metaphase arrest, confluent monolayers of porcine TSCs were treated with 10 g/mL colcemid (Gibco, Carlsbad, CA, USA). Karyotyping analysis was performed as previously described [72]. Chromosomes on the slides were counted and observed under a bright-field microscope to check for cytogenetic abnormalities.

### Statistical analyses

Statistical analyses were performed using GraphPad Prism 8.0. Results are expressed as the mean ± SEM. Experiments were repeated at least three times unless otherwise stated in the legend. Statistical tests were performed using an unpaired two-tailed Student’s t-test or ANOVA. P-values < 0.05 were considered statistically significant. The statistical methods, p-values, and sample numbers are indicated in the figure legends.

### Electronic supplementary material

Below is the link to the electronic supplementary material.

Supplementary Material 1

Supplementary Material 2

Supplementary Material 3

Supplementary Material 4

## Data Availability

The datasets used and/or analyzed during the current study are available from the corresponding author upon reasonable request.

## Abbreviations

AP: Alkaline phosphatase
BCIP: 5-bromo-4-chloro-3-indolyl phosphate toluidine salt
cDNA: Complementary DNA
COCs: Cumulus oocyte complexes
DMEM: Dulbecco’s Modified Eagle Medium
EGF: Epidermal growth factor
EMT: Epithelial-mesenchymal transition
ESC: Embryonic stem cell
ICM: Inner cell mass
IF: Immunofluorescence
IVF: In vitro fertilization
IVM: In vitro maturation
LE: Luminal epithelium
MEF: Mouse embryonic fibroblasts
mTBM: Modified Tris-buffered medium
NBT: Nitro blue tetrazolium chloride
NT: Nuclear transfer
OPN: Osteopontin
PA: Parthenogenetic activation
PBS: Phosphate-buffered saline
PD: Population doubling
PFA: Paraformaldehyde
PrE: Primitive endoderm
PZM3: Porcine zygote medium 3
ROCK: Rho-associated protein kinase
SCNT: Somatic cell nuclear transfer
TE: Trophectoderm
TGC: Trophoblast giant cell
TGFβ: Transforming growth factor-β
TLH-PVA: Tyrode’s medium containing 0.05% w/v polyvinyl alcohol
TLH: Tyrode’s medium
TSC: Trophoblast stem cell
Wnt: Wing-less/Integrated
