# Supplementary figures and images for: Distinct properties of putative trophoblast stem cells established from somatic cell nuclear-transferred pig blastocysts
Source: Biol Res. 2024 May 30;57:35. doi: 10.1186/s40659-024-00516-y (PMC11137969; doi:10.1186/s40659-024-00516-y)

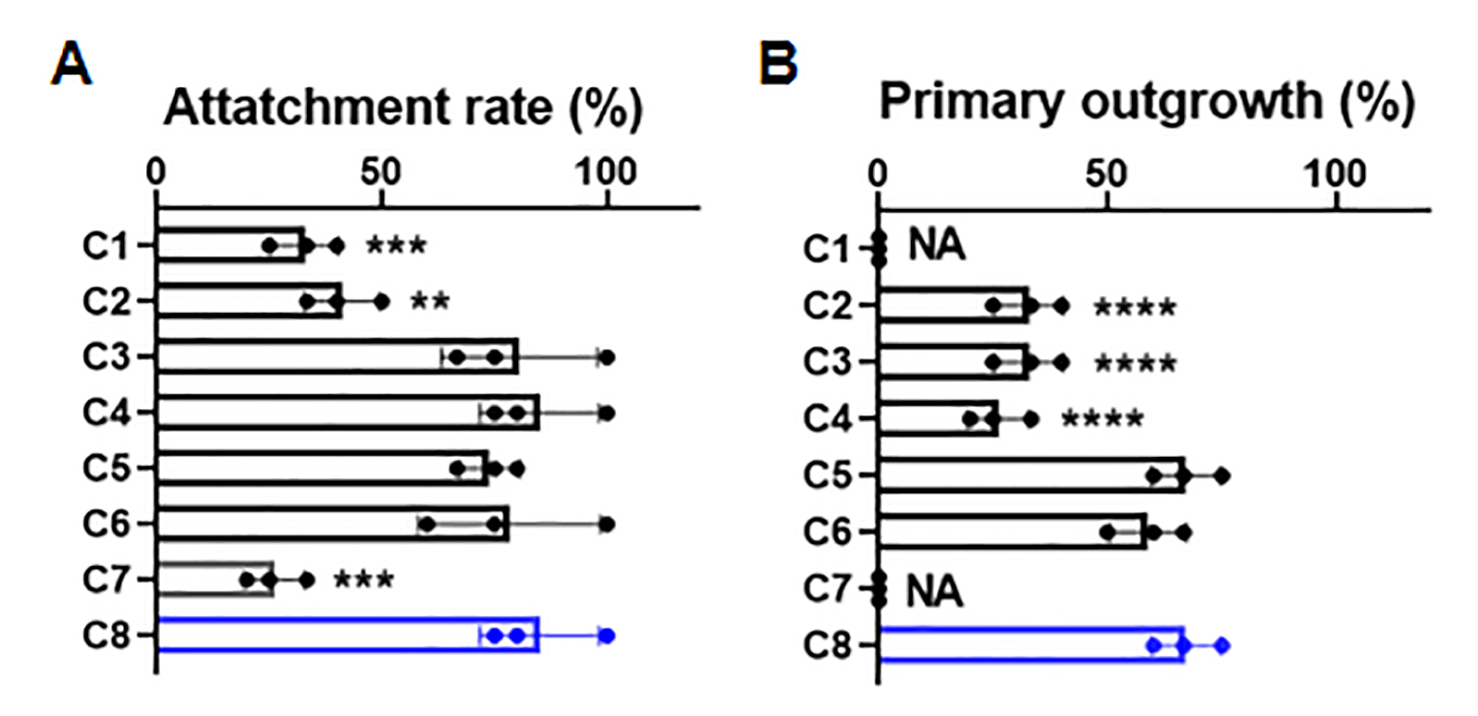

Supplement: Supplementary file 1 — Supplementary Material 1 [file 40659_2024_516_MOESM1_ESM.tif]

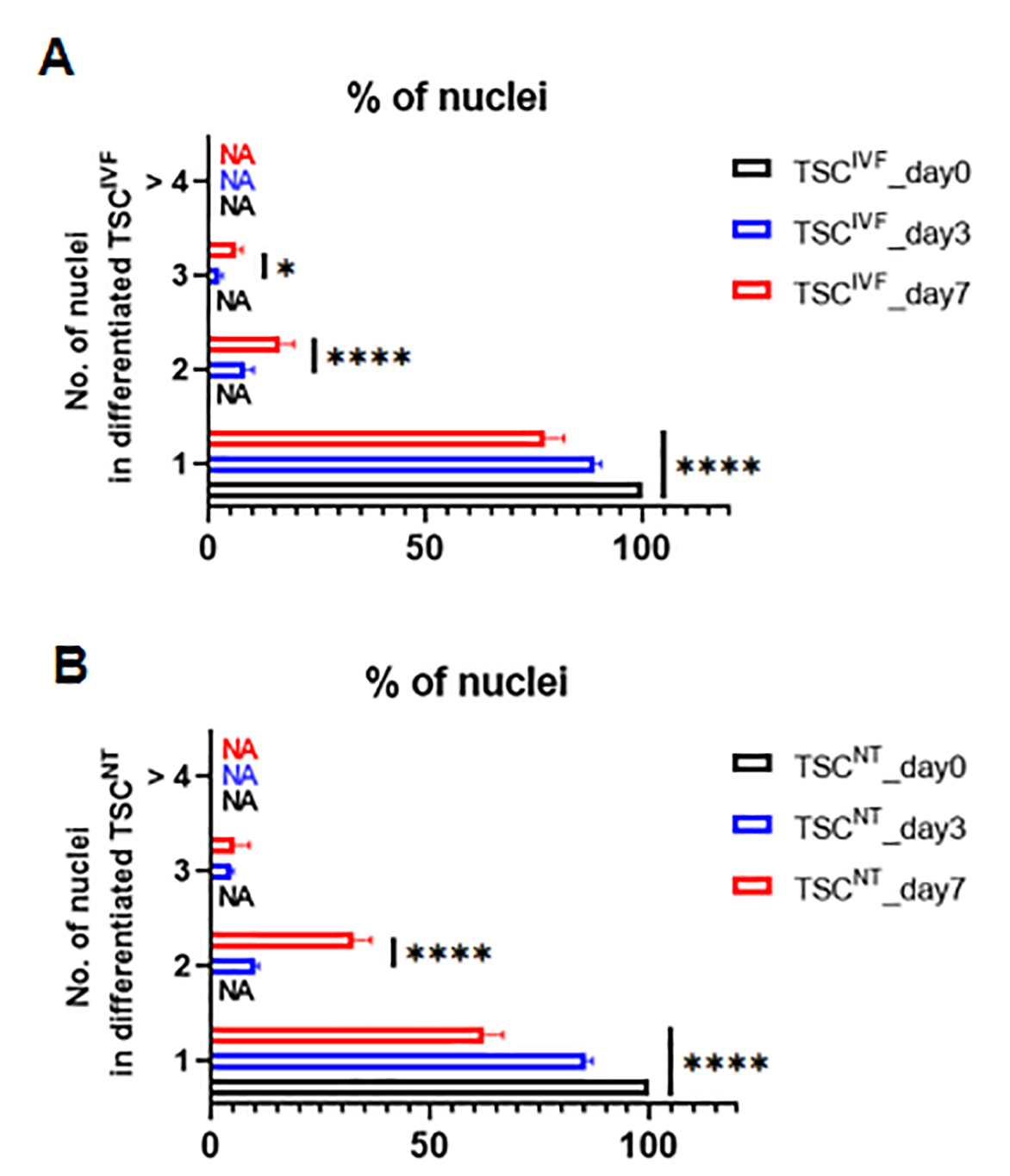

Supplement: Supplementary file 2 — Supplementary Material 2 [file 40659_2024_516_MOESM2_ESM.tif]
