# Supplementary material for: Distinct properties of putative trophoblast stem cells established from somatic cell nuclear-transferred pig blastocysts
Source: Biol Res. 2024 May 30;57:35. doi: 10.1186/s40659-024-00516-y (PMC11137969; doi:10.1186/s40659-024-00516-y)
Supplement: Supplementary file 3 — Supplementary Material 3 [file 40659_2024_516_MOESM3_ESM.docx]

**Table S1. List of primers used for gene expression analysis**

| Genes | Primer sequences | Product size (bp) | Gene Bank  accession number |
| --- | --- | --- | --- |
| *RN18S* | F: 5′-CGCGGTTCTATTTTGTTGGT-3′ | 219 | NR_046261 |
|  | R: 5′-AGTCGGCATCGTTTATGGTC-3′ |  |  |
| *NONOG* | F: 5′-TAAAACCACTGCCCACATCT-3′ | 131 | NM_001129971 |
|  | R: 5′-CTGCCTCTGAAATCTGTCGT-3′ |  |  |
| *GATA6* | F: 5′-GAGGGAATTCAGACCAGGAA-3′ | 159 | NM_214328.2 |
|  | R: 5′-AGCTGGCGTTTGTGTTGTAG-3′ |  |  |
| *TEAD4* | F: 5′-GATCAGGCAGCTAAGGACAA-3′ | 87 | NM_001142666 |
|  | R: 5′-TTTATTGTGGAAGGCTGAGG-3′ |  |  |
| *ITGB6* | F: 5′-GCTCGGTACACATCTGGTCA-3′ | 163 | XM_021074454 |
|  | R: 5′-GGGCCTCAGTCAGGAATGTA-3′ |  |  |
| *YBX2* | F: 5′-TCCTCCCCTTTCCCATAATC-3′ | 187 | XM_021067811 |
|  | R: 5′-GTTCCTTCTCAGCCTGATCG-3′ |  |  |
| *VEGFA* | F: 5′-GCCTTGCTGCTCTACCTCCA-3′ | 271 | NM_214084 |
|  | R: 5′-TGGCGATGTTGAACTCCTCAGT-3′ |  |  |
| *CYP11A1* | F: 5′-GTCCCATTTACAGGGAGAAGCTCG -3′ | 182 | NM_214427 |
|  | R: 5′-GGCTCCTGACTTCTTCAGCAGG-3′ |  |  |
| *CYP19A1* | F: 5′-GCATCATGCTGGACACCTCT -3′ | 101 | NM214429 |
|  | R: 5′-AGCTTGCCATGCATCAAAAT -3′ |  |  |
| *HSD3B1* | F: 5′-CTCCGTGGTCATCCACACTG-3′ | 164 | NM_001004049 |
|  | R: 5′-GTCCAGCCACCTCTATGCTG -3′ |  |  |
| *HSD11B2* | F: 5′-GGTCAAGGTCAGCGTCATCCA-3′ | 231 | NM_213913 |
|  | R: 5′-GCCAGCAGTGCGTCAGTGAT-3′ |  |  |
| *SFMBT2* | F: 5′-GACCAAAGAGATGCGTCAGA-3′ | 178 | NM_001014428 |
|  | R: 5′-CTTTGGACTCTCACGACGAA-3′ |  |  |
| *SLC38A4* | F: 5′-TCTTCACGGCAGTGGAGTAG-3′ | 111 | XM_021092580 |
|  | R: 5′-GCAAATGCTAGGATGGGAAT-3′ |  |  |
| *BAX* | F: 5′-TGCCTCAGGATGCATCTACC-3′ | 199 | XM_003127290 |
|  | R: 5′-AAGTAGAAAAGCGCGACCAC-3′ |  |  |
| *BCL2* | F: 5′-AATGACCACCTAGAGCCTTG-3′ | 182 | NM_214285 |
|  | R: 5′-GGTCATTTCCGACTGAAGAG-3′ |  |  |

F: Forward, R: Reverse
