# Supplementary material for: Distinct properties of putative trophoblast stem cells established from somatic cell nuclear-transferred pig blastocysts
Source: Biol Res. 2024 May 30;57:35. doi: 10.1186/s40659-024-00516-y (PMC11137969; doi:10.1186/s40659-024-00516-y)
Supplement: Supplementary file 4 — Supplementary Material 4 [file 40659_2024_516_MOESM4_ESM.docx]

**Table S2. List of antibodies used for immunofluorescence staining**

| Detection of | Name | Host species | Dilution or Concentration | Manufacturer (Cat #) |
| --- | --- | --- | --- | --- |
| Trophoblast stem cell | CDX2 | Mouse IgG1 | 1:200 | Biogenex  (MU392A-5UC) |
| Trophoblast stem cell | Cytokeratin7 (KRT7) | Mouse IgG1 | 1:200 | Abcam  (ab9021) |
| Epithelial cell | E-cadherin (CDH1) | Rabbit IgG | 1:200 | Cell Signaling  (3195) |
